# Case Report: Empyema secondary to percutaneous transthoracic needle biopsy in three cases

**DOI:** 10.3389/fmed.2025.1531909

**Published:** 2025-05-16

**Authors:** Rao Du, Hui Mao, Wei Min Li, Dan Liu, Kaige Wang

**Affiliations:** ^1^Department of Radiology, West China Hospital, Sichuan University, Chengdu, China; ^2^Department of Pulmonary and Critical Care Medicine, West China Hospital, Sichuan University, Chengdu, China

**Keywords:** empyema, percutaneous transthoracic needle biopsy, lung abscess, medical thoracoscopy, metagenomic next-generation sequencing

## Abstract

Percutaneous transthoracic needle biopsy (PTNB) is a widely utilized diagnostic procedure for pulmonary lesions, with the current literature predominantly documenting pneumothorax and hemorrhage as primary complications. While empyema represents a rare complication, its clinical implications warrant special attention. In this study, we report the cases of three patients with unidentified pulmonary masses who developed empyema after PTNB. All cases exhibited fever (24–48 h post-procedure) and radiographic evidence of pleural effusion progression shortly after the procedure, which was successfully managed through pleural drainage and antibiotic treatment. These findings suggest that pre-procedural infectious foci may be prone to iatrogenic pleural seeding during PTNB. This report emphasizes the necessity of monitoring infectious indicators in patients undergoing biopsy of cavitary or necrotic lesions. Physicians should exercise caution when puncturing lumps suspected of abscesses and remain vigilant for empyema secondary to PTNB if the patient shows signs of infection.

## Highlights

- Comprehensive radiologic assessment of pulmonary masses with air-fluid levels and cavity is crucial before percutaneous transthoracic needle biopsy.
- Selecting the appropriate puncture path, preferentially entering the needle from the anterior adhesion, and using a coaxial puncture needle to avoid pleural injury from repeated punctures may help reduce the risk of empyema secondary to PTNB.
- If a patient presents with signs of infection after a percutaneous transthoracic needle biopsy, physicians should remain vigilant for the development of empyema.
- Empyema is usually caused by multiple pathogens. Anaerobic organisms cannot be cultured by the conventional method. mNGS is a useful method for difficult-to-culture pathogens.
- Medical thoracoscopy can promote local drainage in patients with empyema.

## Introduction

Computed tomography (CT)-guided percutaneous transthoracic needle lung biopsy (PTNB) has emerged as the preferred diagnostic procedure for pulmonary masses, offering a less invasive approach with reduced complications. It has been used since the 1960s (1) and is becoming more important in the era of CT screening for lung cancer and precision medicine. Common complications include pneumothorax, pulmonary hemorrhage, systemic air embolism, and tumor seeding (2). Cases of empyema following PTNB have been rarely reported (3). In this study, we present three cases of empyema that occurred in patients after PTNB. Our aim is to enhance the consciousness regarding empyema following PTNB among clinicians and highlight the importance of detailed pre-procedural evaluation.

## Case presentation

### Case 1

**Figure 1:**
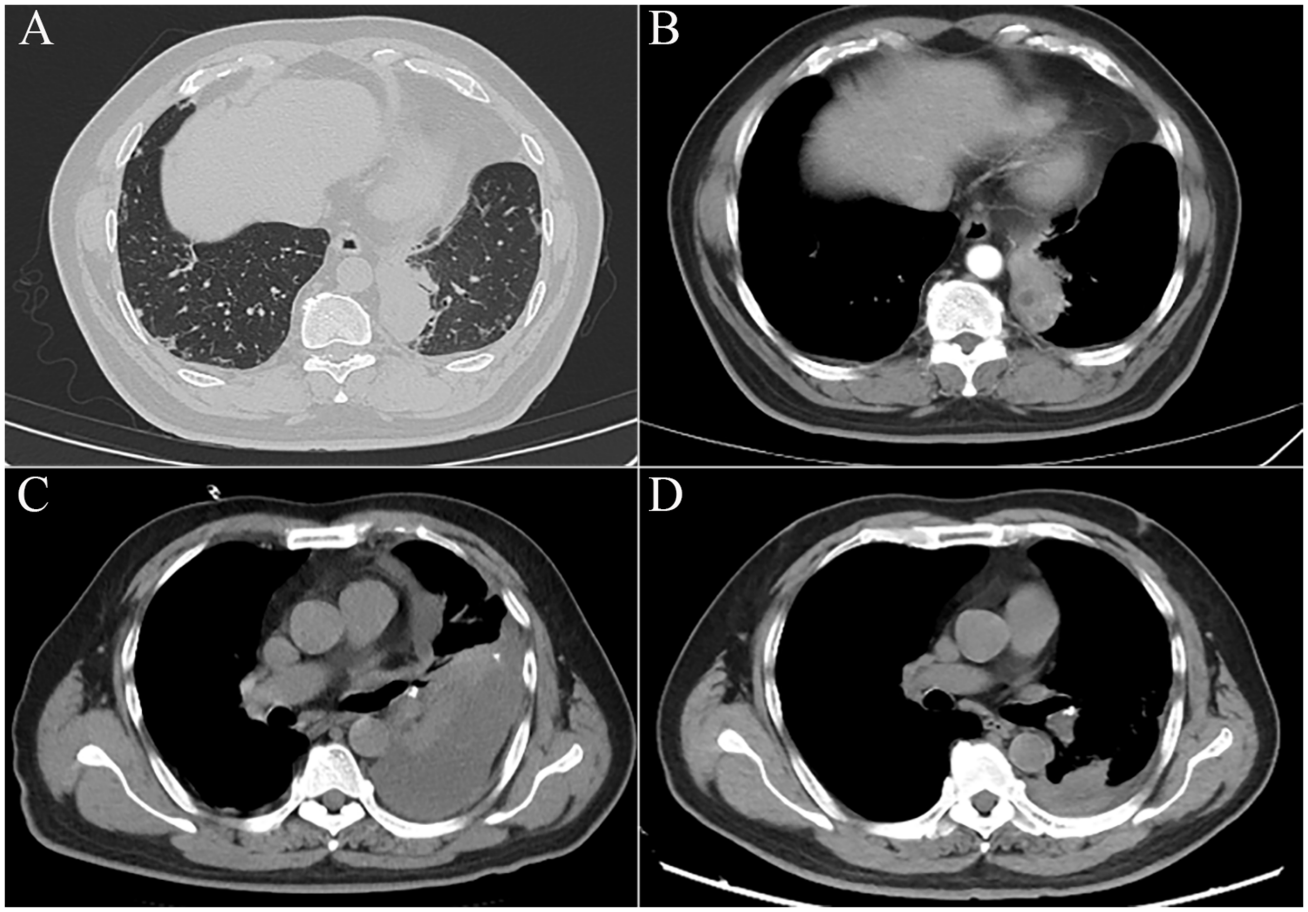
**(A)** Soft tissue shadow adjacent to the mediastinum in the inferior lobe of the left lung, approximately 5.6 × 3.7 cm in its maximum cross-section. **(B)** The mass was enhanced unevenly, with small areas of cystic necrosis. **(C)** Chest CT scan revealed a substantial accumulation of effusion in the left thorax after PTNB within 12 h. **(D)** Two days before discharge, a chest CT scan indicated pleural effusion had been absorbed significantly.

A 56-year-old man was referred to our hospital as he had been experiencing persistent cough for 2 months and hemoptysis for the past 7 days. A chest CT scan revealed the presence of a soft tissue shadow adjacent to the mediastinum in the inferior lobe of the left lung (Figure 1A). The contrasted scan showed an uneven enhancement with small areas of cystic necrosis inside the pulmonary mass (Figure 1B). Based on these findings, the possibility of neoplastic or infective lesions was considered. The tests of peripheral blood were normal except for a slight increase in C-reactive protein (CRP) with 14.4 mg/L (normal range, NR: 0–6 mg/L). Despite undergoing 13 days of antibacterial treatment with piperacillin-sulbactam, the patient’s condition did not improve. Therefore, PTNB was performed. Subsequently, the patient experienced a fever with a maximum temperature of 38.9°C after 10 h, accompanied by pronounced chest pain. Laboratory findings revealed elevated white blood cell count of 18.74 ×10^9^/L (NR: 0–10 ×10^9^/L), percentage of neutrophils (85.9%), procalcitonin (PCT, 1.53 ng/mL), and CRP (143 mg/L) levels in the blood. A timely chest CT scan revealed a substantial accumulation of effusion in the left thorax (Figure 1C). Thoracic drainage revealed yellow, turbid pleural fluid. The pleural fluid test results showed the presence of 27,000 ×10^6^/L nucleated cells, of which 97% were multinucleated. In addition, a glucose concentration of 1.47 mmol/L and a lactate dehydrogenase (LDH) level of 1,640 IU/L were noted in the pleural fluid. Both the smear and culture were negative. The lung tissue exhibited chronic inflammation with no evidence of neutrophil infiltration. The presence of histiocytic cells and newly forming granulation tissue in the alveolar cavities indicated the development of organizing pneumonia. Furthermore, there were no fungi or tuberculosis. The symptoms, including chest pain, cough, and sputum, were improved, and the temperature gradually returned to normal after 4 days. The repeated white blood cell (WBC) count, CRP, and PCT levels decreased to within the normal range. Two days before discharge, a repeated chest CT scan indicated that pleural effusion had been absorbed significantly (Figure 1D).

### Case 2

**Figure 2:**
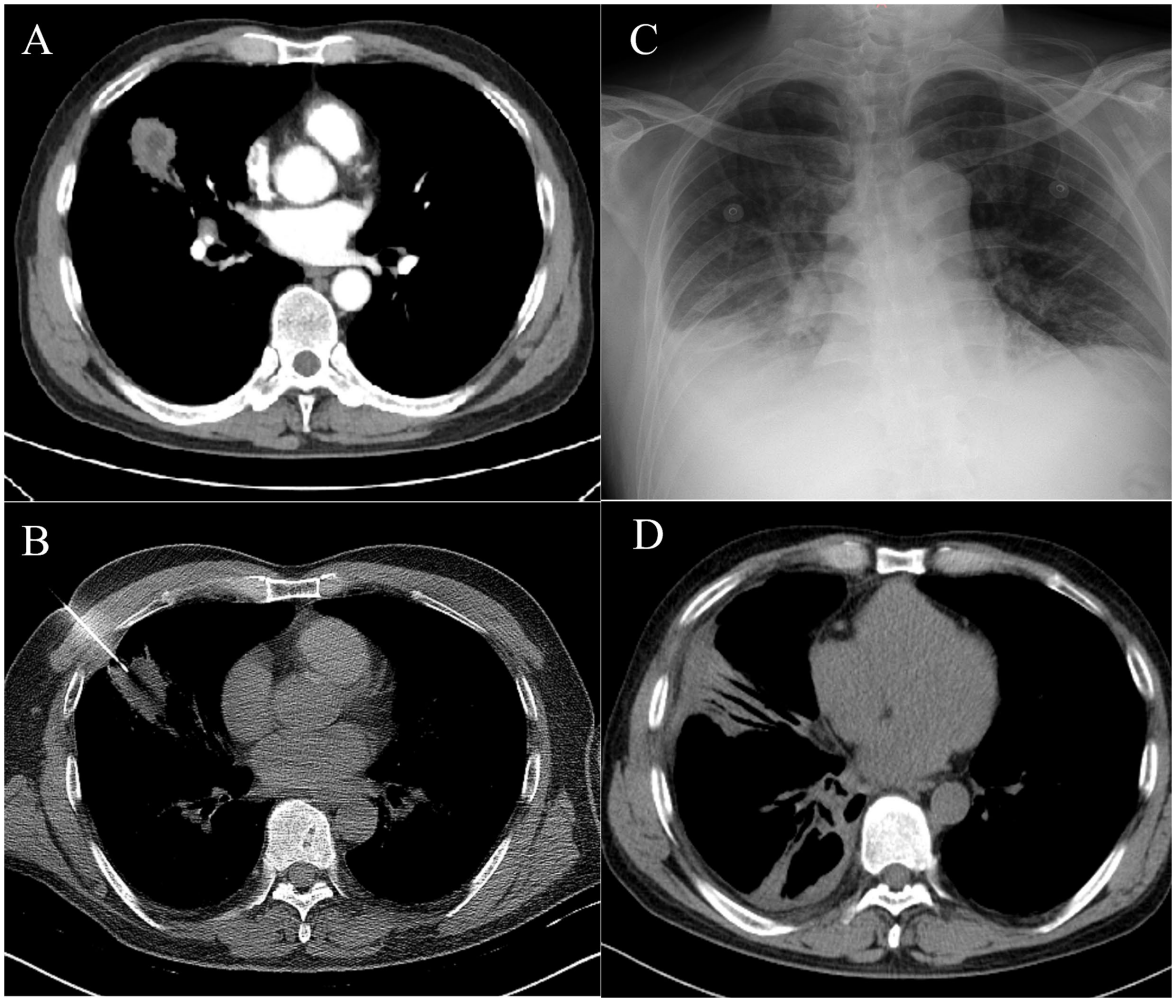
**(A)** Chest CT showed an irregular soft tissue mass of approximately 4.2 cm × 4.1 cm in the right lung, with a lobulated and blurred edge. Enhanced CT showed a low-density shadow within the mass. **(B)** PTNB of the right lung mass. **(C)** The chest x-ray showed pleural effusion in the right thorax. **(D)** The follow-up chest CT showed significant absorption of the lesions.

The patient, a 52-year-old man with 4 months of cough and expectoration, was admitted to our hospital. Routine laboratory tests revealed an elevated level of CRP (18.5 mg/L), a normal range of WBC, neutrophil proportion, and PCT. A subsequent chest CT scan demonstrated the presence of an irregular soft tissue mass measuring approximately 4.2 cm × 4.1 cm in the right lung, which exhibited a lobulated contour and a blurred edge. Enhanced CT scan showed the presence of a low-density shadow within the mass (Figure 2A). Further characterization of the lesion was sought, leading to the recommendation of PTNB as a diagnostic modality. Informed consent was obtained from the patient, and the procedure was performed on the third day of hospitalization (Figure 2B). Post-procedure, the patient experienced a fever of 38.6°C within 12 h, accompanied by worsening right chest pain with reduced respiratory sounds. WBC increased to 12.3*10^9^/L, with neutrophils accounting for 80.5% of the total count. Moreover, both PCT and CRP levels were significantly elevated. PCT surpassed the normal range by more than 60-fold (1.26 ng/mL), while CRP was measured at 158 mg/L. In addition, a chest x-ray revealed a massive pleural effusion (Figure 2C), and a chest ultrasound suggested a viscous pleural effusion in the right thoracic cavity. Thoracocentesis was performed, draining turbid and yellow pleural effusion. The physicochemical properties of the pleural fluid revealed that it was exudative, containing a total of 8,000*10^6^/L of nucleated cells, 77% of which were multinucleated cells. Additionally, the glucose level was measured at 2.1 mmol/L, and the LDH level was 881 IU/L. The smear and culture were negative. Meanwhile, imipenem-cilastatin sodium treatment was administered. Subsequently, the patient’s fever subsided after 3 days, and his clinical condition improved significantly. The pathological results revealed infiltration of inflammatory cells and no evidence of malignant cells. Before the day of discharge, the WBC, CRP, and PCT decreased to the normal range, the chest CT showed that the lesions absorbed (Figure 2D).

### Case 3

**Figure 3:**
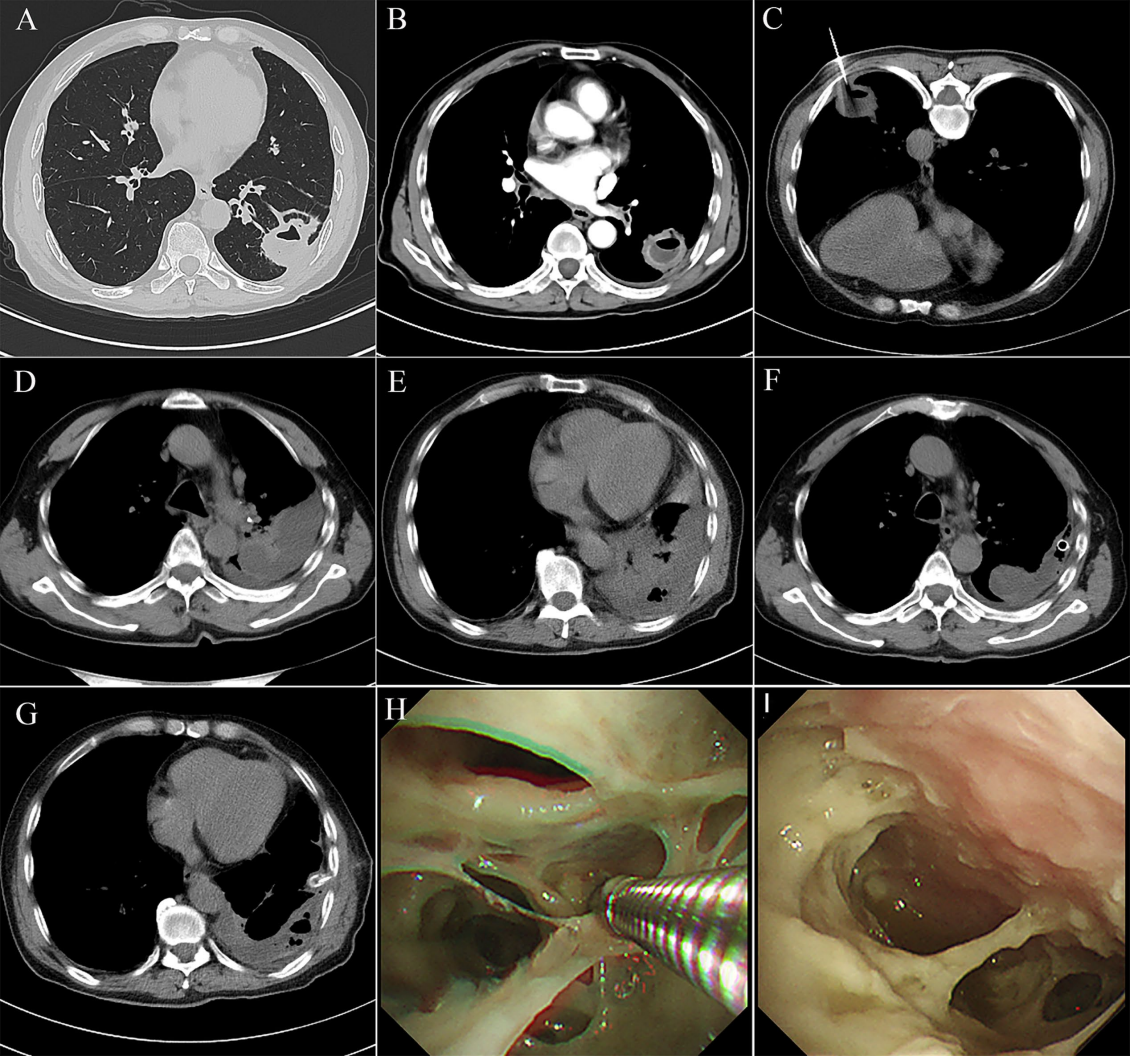
**(A)** Chest computed tomography showed an irregular soft tissue mass of approximately 3 cm*2.6 cm, located in the outer basal segment of the lower lobe of the left lung, with displayed lobes, spicules, local pleural thickening, and adhesion. **(B)** Contrasted CT showed the edge of the mass was significantly enhanced, with an air-fluid level. **(C)** A PTNB was performed on the pulmonary mass. **(D,E)** There were amounts of pleural effusion in the left thorax. **(F,G)** Medical thoracoscopy revealed a significant amount of viscous, purulent fluid and a variety of abscess cavities. **(H,I)** Follow-up chest CT showed a significant absorption of the pleural effusion.

A 67-year-old man presented with persistent cough and expectoration for 1 month, no fever, dyspnea, and chest pain or distress. The patient had underlying diseases, including diabetes mellitus and hypertension, for 2 years. He had a history of smoking and alcohol consumption for more than 30 years, but he quit smoking 3 years ago. Meanwhile, his tooth surfaces were decayed. Laboratory routine tests are shown in Table 1. A subsequent CT chest scan revealed an irregular soft tissue mass in the lower lobe of the left lung (Figure 3A), and the contrast-enhanced CT showed that the edges were significantly enhanced and the presence of an air-fluid level within it (Figure 3B). Despite receiving broad-spectrum antibiotics for over 2 weeks, there was no improvement. As a result, lung cancer was suspected. Then, PTNB was performed on the third day of administration (Figure 3C) to identify the presence of either a tumor or an infection. The process went smoothly. However, the patient developed a fever with a maximum temperature of 39°C and chills after 6 h of PTNB. No pathogen was found in the blood cultures of the patient. A subsequent chest CT scan revealed an increased amount of pleural effusion in the left thorax (Figures 3D,E). Administration of imipenem-cilastatin sodium and drainage were initiated immediately. Thoracic drainage revealed yellow turbid pleural fluid, which was found to contain purulent cells. The feature of pleural fluid is shown in Table 1. Symptomatic improvement was noted in the patient, and temperature gradually decreased to a normal level. Metagenomic next-generation sequencing (mNGS) of the pleural effusion included mixed anaerobic bacteria. The top four genera identified were *Prevotella, Streptococcus, Peptostreptococcus,* and *Parvimonas*, with 1,257,808, 209,457, 197,966, and 106,140 reads, respectively. At the species level, *Prevotella oris* had 684,013 reads, *Streptococcus constellatus (S. constellatus)* had 82,594 reads*, Peptostreptococcus stomatis* had 194,189 reads, and *Parvimonas micra* had 106,140 reads. The clinical physician connected with the laboratory physician immediately. *S. constellatus* was cultured from the pleural fluid through the modified culture method. The lung tissue exhibited chronic inflammatory changes with no evidence of neutrophil infiltration. The presence of histiocytic cells and newly forming granulation tissue in the alveolar cavities indicated organizing pneumonia. Furthermore, there were no fungi or tuberculosis. After 7 days, the daily output of the tube was less than 100 mL. Chest ultrasonography was performed, and there were variably sized pus cavities in the left thorax. To enhance drainage, a medical thoracoscopy was conducted, revealing a significant amount of viscous, purulent fluid and a variety of abscess cavities (Figures 3F,G). The patient recovered gradually; symptoms such as cough, chest pain, and sputum improved. WBC, CRP, and PCT decreased to the normal range. The follow-up chest CT also confirmed improvement in the patient’s condition (Figures 3H,I).

**Table 1:** Laboratory test of case 3.

Serum	Before PTNB	6 h after PTNB	1 day after medical thoracoscopy
WBC (NR: 3–10*10^9^/L)	7.9	13.74	9.7
NEUT (NR: 40–75%)	65.4	83.1	74.7
PCT (NR: <0.046 ng/mL)	0.57	5.33	1.02
CRP (NR: <5 mg/L)	62	313	89
IL-6 (NR: 0–7.00 pg./mL)	14.8	234	83.7
Total protein (NR: 65–85 g/L)	64.2	60.7	57.6
protein (NR: 40–55 g/L)	34.7	33.5	33.0
LDH (NR: 120–250 IU/L)	112	289	155
Pleural effusion			
Color	Yellow and turbid		
Pus	Positive		
Nucleated cells (10^6^/L)	30,000		
Multinucleated cells (%)	97		
Total protein	48.3		
Protein	26.6		
ADA	32.3		
LDH	2,267		
Glucose	0.04		
Cl	94.4		
Smear	G^+^ coccus, G^+^ /G^−^ bacilli		
Culture	*Streptococcus constellatus*		

NR, normal range; WBC, white blood cell; NEUT, neutrophils; PCT, procalcitonin; CRP, C-reactive protein; IL-6, interleukin 6; LDH, lactate dehydrogenase; ADA, adenosine deaminase.

## Discussion

For pulmonary mass, imaging techniques alone are often inadequate to differentiate benign lesions from the malignant ones or to define their histological characteristics. In the majority of cases, a pulmonary nodule requires a pathological analysis, which should be obtained with the safest and least invasive method that still ensures that the collection of tissue samples is sufficient for histological and molecular analyses (2). PTNB is a useful modality to offer a less invasive approach with reduced complications. It can serve as a valuable tool for identifying the causative organism in cases where bronchoscopy fails to provide a definitive diagnosis or when thoracic lesions are not suitable for bronchoscopy (4), especially for the peripheral and subpleural lesions of the lung (5). In our three cases, the possibility of malignancy of the pulmonary mass cannot be ruled out because of the lack of improvement after a relatively long time of broad-spectrum antibiotics, and the masses were located in the peripheral area of the left lung, which was difficult for bronchoscopy; thus, PTNB was performed.

PTNB is generally safe but not risk-free. There are four common complications, including pneumothorax, pulmonary hemorrhage, systemic air embolism, and tumor seeding (6). Empyema is a frequently occurring complication of pneumonia (7), but there have been few reports of empyema caused by PTNB. Empyema, defined as the presence of infected pleural fluid or pus in the pleural space, can arise as a result of pneumonia, thoracic surgery, or chest injury. In the United States and UK combined, it is estimated that empyema affects up to 80,000 individuals per year, carrying a mortality rate of up to 20% (8). Prompt identification and diagnosis of empyema are essential to achieve favorable outcomes. The confirmation of empyema entails the presence of gross aspiration of pus, positive culture, or gram stain. Initially, these effusions present as simple exudative parapneumonic fluid collections, but they progress to become increasingly acidotic, infected, and complex, characterized with an enlarging, loculated effusion with the development of a thick exudate (8).

Infection complications can arise from various factors, including the introduction of infectious agents during the procedure or secondary to the contiguous spread from an infection (9). Our three cases were considered to have empyema caused by PTNB based on the following reasons: (1) radiographic evidence of the progression of the pleural effusion shortly after the procedure, (2) temporal relationship between infection and the procedure, and (3) the features of the pleural fluid. Except for that the abovementioned reasons, the patient’s changed symptoms and response to treatment also support the diagnosis of empyema following PTNB. Why did the three patients develop empyema? We retrospectively analyzed the chest CT of our three cases and concluded that there were necrotic foci inside all the lung masses. We believe that PTNB caused the propagation of preexisting infection from pulmonary lesions to the thoracic cavity because of the penetration of the visceral pleura. Kim, Junghoon et al. reported that CNB had a higher overall complication rate than FNA (52% vs. 20%) (10). Shin, Yoon Joo et al. reported that the complication rate of CT-guided PTNB may be high when the needle passes through honeycomb lesions (11). Careful chest CT evaluation to identify and avoid pre-existing necrotic lung lesions in patients with abscesses may reduce the complication of empyema. In addition, selecting the appropriate puncture path, preferentially entering the needle from the anterior adhesion, and using a coaxial puncture needle to avoid pleural injury caused by repeated puncture can reduce the empyema secondary to PTNB.

The management of empyema necessitates the administration of appropriate antibiotics and the implementation of drainage (12). Early and adequate drainage of the infected pleural effusion is crucial for successful treatment. Sole reliance on thoracentesis is insufficient and thus not recommended (13). The selection of appropriate antibiotics holds paramount significance. The etiology of empyema has been confirmed to be a mixed infection involving various pathogens, including anaerobic organisms (14). Empirical antibiotic therapy should cover community-acquired bacteria, such as *Streptococcus* spp., *Staphylococcus aureus*, and *Escherichia coli*, and anaerobic organisms primarily, such as *Bacteroides* spp.*, Peptostreptococcus* spp., and *Fusobacterium* spp. It is recommended to administer a parenteral second-generation or third-generation cephalosporin, such as ceftriaxone, in combination with metronidazole, or an aminopenicillin and beta-lactamase inhibitor combination, such as ampicillin-sulbactam (13).

Initially, our two patients (cases 1 and 2) did not exhibit significant systemic inflammatory responses. Based on this observation, we speculated a possible diagnosis of chronic lung abscess. It is well-known that both anaerobic and aerobic bacteria can cause lung abscess, but anaerobic bacteria are generally more common, and the majority of them are polymicrobial. Isolating anaerobic bacteria in primary lung abscess is challenging due to the contamination of respiratory tract specimens by upper airway flora, making them unsuitable for anaerobic culture (7). In cases of pleural infection, it has been reported that approximately 80% of pleural fluid cultures yield negative results in conventional Gram stain and culture. Studies have shown that the utilization of blood culture bottles for culturing pleural fluid can enhance the detection of microorganisms (15). The utilization of a standard culture instead of blood culture bottles may be one of the reasons for the negative culture in two of our cases. Our patient in case 3 developed suppurative inflammation as a complication of PTNB after 6 h. Purulent pleural fluid was drained, and the culture was identified as *S. constellatus.* mNGS for the pleural fluid concluded a variety of anaerobic bacteria. Therefore, we hypothesize that the lung abscess was caused initially by *S. constellatus* or a mixed microorganism.

*S. constellatus,* a gram-positive and catalase test-negative coccus, is known for causing pyogenic infections at various anatomical locations (16). In our case, it may cause a lung abscess first and then spread to the thorax, causing empyema following PTNB. However, the detection of *S. constellatus* can pose challenges when relying on routine clinical laboratory tests, as its preference for 5% CO2 and prolonged anaerobic culture conditions can be relatively insensitive and time-consuming (17). In the case of our patient, the negative sputum culture may be attributed to the limitations of routine laboratory culture methods, which may not include anaerobic culture or allow for prolonged culture periods.

mNGS is an unbiased approach that can theoretically detect all pathogens in one clinical sample. For difficult-to-culture pathogens, such as *S. constellatus,* mNGS is a useful method (17). Primary lung abscesses and empyema are often polymicrobial and contain multiple anaerobic species (18). In our case, mNGS not only detected *S. constellatus* but also a variety of anaerobic bacteria which may originate from the oral cavity. The results of mNGS were sent to the physician in less than 24 h, facilitating prompt communication between the clinical physician and laboratory physician. *S. constellatus* was successfully cultured under anaerobic conditions. In clinical practice, it is essential to enhance timely communication between clinical physicians and laboratory physicians.

Early stratification of patients with empyema with multiloculated pleural effusion using medical thoracoscopy can lead to better outcomes (19). Studies have shown that medical thoracoscopy can be well tolerated and consistently effective, with success rates ranging between 73 and 100% (20). Especially, it is possible to open multiple loculations, drain purulent liquid, remove fibrous adhesions, and apply local fibrinolytics. The patient in case 3 developed pus cavities, and a timely medical thoracoscopy was performed, resulting in a favorable outcome.

## Conclusion

In conclusion, the careful pre-procedural evaluation of pulmonary mass with an air-fluid level is crucial before PTNB. Selecting the appropriate puncture path, preferentially entering the needle from the anterior adhesion, and using a coaxial puncture needle to avoid pleural injury from repeated punctures may help reduce the risk of empyema secondary to PTNB. Additionally, if a patient presents with signs of infection after PTNB, physicians should remain vigilant for the development of empyema. Empyema is often caused by multiple pathogens, and anaerobic organisms cannot be cultured by conventional methods. Thus, mNGS is a useful method for identifying difficult-to-culture pathogens. Medical thoracoscopy can promote local drainage in patients with empyema.

## Data Availability

The original contributions presented in the study are included in the article/supplementary material, further inquiries can be directed to the corresponding author.
